# A high-performance molecular switch fabricated by the two-dimensional van der Waals heterojunction

**DOI:** 10.1093/nsr/nwad131

**Published:** 2023-05-09

**Authors:** Xubin Zhang, Adila Adijiang, Dong Xiang

**Affiliations:** Institute of Modern Optics and Center of Single-Molecule Science, Tianjin Key Laboratory of Micro-scale Optical Information Science and Technology, Nankai University, China; Institute of Modern Optics and Center of Single-Molecule Science, Tianjin Key Laboratory of Micro-scale Optical Information Science and Technology, Nankai University, China; Institute of Modern Optics and Center of Single-Molecule Science, Tianjin Key Laboratory of Micro-scale Optical Information Science and Technology, Nankai University, China; TongLu FeiRan Garment-Nankai University Joint Laboratory, Nankai University, China; School of Materials Science and Engineering, Smart Sensing Interdisciplinary Science Center, Nankai University, China

Since the concept that single molecules can be used as electronic components to construct active electronic devices was proposed, the preparation of commercially available single-molecule switches has become a research hotspot. To break through the dilemma of electronic components facing the failure of Moore's Law and further reduce the size of a chip, developing single-molecule electronics is particularly important and has yielded a wealth of results. Many research groups have successfully used optical fields [1,2], electric fields [3–5], and electrochemical methods [6] to modulate single-molecule behaviors and achieve single-molecule switching properties. Although the techniques for modulating single-molecule conductance have matured, it is still difficult to achieve molecular switches with consistently higher switching ratios and smaller sizes. In the traditional mechanically controllable break junction (MCBJ) and the scanning tunneling microscope break junction (STM-BJ) techniques, electrical characterizations are influenced by the molecular length and anchoring groups, so the design of single-molecule junctions is usually limited in terms of the size of the junction and the synthesis steps of the molecule, making it difficult to design more functional units in molecular switches.

**Figure 1.**
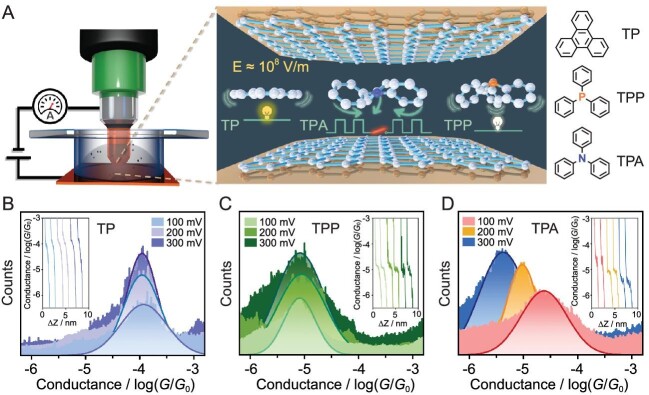
Electrical characterization of M-2D-vdWHs. (A) Schematic diagram of the XPBJ setup and the conformational evolution of three M-2D-vdWHs under the applied electric field. Right: The chemical structures of triphenylene (TP), triphenylphosphine (TPP), and triphenylamine (TPA) were used to fabricate M-2D-vdWHs. (B to D) The 1D conductance histograms for TP, TPP and TPA M-2D-vdWHs under the bias of 100 mV, 200 mV and 300 mV, respectively. The inserts give the typical conductance-displacement traces. The figure is adapted from Ref. [7].

To solve these difficulties, Yang and co-workers developed a new cracking technology called cross-plane break junction (XPBJ) system, to prepare a single-molecule 2D van der Waals heterojunction (M-2D-vdWH) [7], as presented in Fig. 1. The method of using the van der Waals force to bridge single molecules between electrodes to form a single molecular junction resolves the issue that the size of the single molecular junction is limited by the length of the single molecule, and that both ends of the single molecule have to be modified with anchoring groups. The highlights of this article are as follows:

Employing the XPBJ technique, a graphene-covered copper substrate is used as the bottom electrode, and the top electrode is made by bending a copper wire covered with graphene. Piezoelectric stacks control the movement of the top electrode through the current feedback when the width of the nanogap between the top and bottom electrodes matches the thickness of the molecule by setting an upper limit of conductance to be 10^–2.80^*G*_0_ (*G*_0_ = 2e^2^/h, quantum conductance) in the current feedback process; an M-2D-vdWH is fabricated due to the van der Waals force between the molecule and the graphene. The configuration of the heterojunction is more stable than the traditionally molecular break junction structure.

Compared with the way of using single-molecule anchoring groups to connect electrodes to form a single-molecular junction, Yang's team used intermolecular van der Waals forces to sandwich triphenylamine (TPA) molecules between two layers of graphene to form a single-molecular junction. In measuring the width of the TPA single-molecular junction, they derived a distance between the electrodes of 1.2 nm, a value smaller than the theoretically calculated molecular length (1.6 nm) and closer to the thickness of the molecule (0.96 nm) with the cross-plane charge transport mode. With the aid of Raman spectroscopy, and in conjunction with two previous studies [8,9], it was effectively proved that in M-2D-vdWH the molecule is sandwiched between two electrodes, i.e. the thickness of the molecule determines the length of the molecular junction, rather than the previously generally accepted molecular length. Finally, the electric field has the properties of regulating the conformational change of molecules and promoting the catalytic reaction of these molecules [3,10]. In this work, researchers change the bias between 100 mV and 300 mV to control the electric field in the molecular junction in order to regulate the conformation of TPA, and they found that TPA can switch from the three-wing propeller (TWP) conformation (high conductance state, 100 mV, 10^−4.58^*G*_0_) to the triangular cone (TC) (low conductance state, 300 mV, 10^−5.36^*G*_0_) to realize the switching properties of single-molecule junctions (the on–off ratio is approximately 6). On the other hand, by switching back and forth between 100 mV and 300 mV of the applied bias at room temperature, the molecular junction can transition stably between high and low conductance states, which shows that the TPA molecular junction has the potential application for realizing molecular switching at room temperature in a stable manner.

This work will promote the development of single-molecule switches, and the proposed XPBJ and M-2D-vdWH techniques provide new methods for the realization of stable single-molecule switches and the integration of electronic components, enabling the construction of molecular junctions down to the range of atomic thickness. We expect that the M-2D-vdWH technology can be further combined with other regulation methods (e.g. optical fields) to explore other types of single-molecular switching.
